# Residual Compressive Behavior of Self-Compacting Concrete after High Temperature Exposure—Influence of Binder Materials

**DOI:** 10.3390/ma15062222

**Published:** 2022-03-17

**Authors:** Marija Jelčić Rukavina, Ivan Gabrijel, Ivanka Netinger Grubeša, Ana Mladenovič

**Affiliations:** 1 Faculty of Civil Engineering, University of Zagreb, Fra Andrije Kačića Miošića 26, 10000 Zagreb, Croatia; ivan.gabrijel@grad.unizg.hr; 2 Faculty of Civil Engineering and Architecture Osijek, University Josip Juraj Strossmayer of Osijek, Vladimir Prelog Street 3, 31000 Osijek, Croatia; nivanka@gfos.hr; 3 Slovenian National Building and Civil Engineering Institute, Dimičeva ulica 12, 1000 Ljubljana, Slovenia; ana.mladenovic@zag.si

**Keywords:** self-compacting concrete, mineral additives, high temperatures, residual mechanical properties

## Abstract

This paper presents an experimental investigation of the compressive behavior of high-strength self-compacting concrete exposed to temperatures up to 600 °C. Ten different concrete compositions were tested, in which part of the cement (by weight) was replaced by three different mineral additives (5–15% metakaolin, 20–40% fly ash and 5–15% limestone). The stress–strain curves, compressive strength, modulus of elasticity and strain at peak stress were evaluated from uniaxial compression tests. Scanning electron microscope micrographs were also taken to evaluate the damage caused by the high temperatures. A sharp decrease in mechanical properties and an increase in peak strain were observed already after 200 °C for all mixes tested. The different mineral additives used in this study affected the variations of residual compressive strength by 24% and peak strain by 38%, while the variations of residual modulus elasticity were 14%. Comparing the obtained results with the recommendations for compressive strength given in regulatory code EN 1992-1-2 for high strength concrete, it can be concluded that the strength loss observed in EN 1992-1-2 at temperatures up to 400 °C is too conservative. The Popovics model for the relationship between stress and strain provided a good approximation for the experimentally determined stress–strain curves at different temperatures.

## 1. Introduction

Concrete provides the best fire resistance out of all building materials because it is a non-combustible material that does not produce smoke or burning droplets when exposed to fire. However, this does not mean that its properties will remain intact due to fire exposure. The properties that concrete retains after cooling from high temperatures are generally referred to as residual properties and are important in assessing the load-bearing capacity of the fire-affected member [1].

The residual concrete’s properties of greatest interest include compressive strength, tensile strength, modulus of elasticity and stress–strain response [2]. Each of the residual concrete properties is highly dependent on the composition of the concrete mix in question. In addition, the duration of heat exposure, heating regime and the method of cooling affect the residual properties [3,4]. Nevertheless, in the absence of test results, practitioners usually use recommendations from codes, such as EN 1992-1-2 (EC2) [5]. For normal concrete, EC2 [5] provides values for the main parameters of stress–strain relationships as a function of the type of aggregate (siliceous or calcareous) at a given temperature, while for high strength concrete, it provides a reduction in strength depending on three different concrete classes. EN 1994-1-2 (EC4) [6] provides values for the main parameters of the stress–strain relationships of normal (NC) and lightweight concrete (LC) at a given temperature. 

Compared to normal vibrated concrete, self-compacting concrete (SCC) contains a much higher amount/volume of fine particles (≤0.125 mm) needed to achieve mix stability or cohesion. A high percentage of fine particles in SCC is usually achieved by using mineral additives (pozzolanic and non-pozzolanic) such as fly ash, slag, silica fume, limestone, metakaolin, etc., since the use of cement alone as fines results in high production costs [7,8,9]. While the addition of mineral additives usually results in high quality and dense microstructure, which has a positive effect on mechanical properties and durability, this compact microstructure results in adverse effects in the concrete at elevated temperatures, as permeability is reduced so that moisture cannot escape as easily, and high stresses are generated [10,11,12]. 

Extensive research has been carried out to study the effects of the different mineral additives on the mechanical properties of normal and high strength mortar and concrete at and after high temperature exposure [3,13]. In recent years, some studies have been conducted on the effect of different mineral additives, both pozzolanic [14,15,16] and non-pozzolanic [17,18], on the residual properties of SCC. Although fly ash is known to have a positive effect on the residual properties of normal concrete [19], contradictory results have been obtained in the available literature for the use of fly ash in SCC. Abed et al. [14] replaced cement mass by fly ash in amounts of 15% and 30% and concluded that the optimum amount of fly ash that does not affect the 15% fire resistance of concrete for temperatures up to 800 °C. Anand et al. [20] obtained significantly lower residual strengths of SCC with fly ash after heating to 900 °C and attributed this to the extremely high fly ash content (40% per cement mass). Uysal and Tanyildizi [21] studied the effects of various mineral additive after heating to 800 °C, including fly ash up to 35% of the cement replacement and limestone filler up to 30% on the residual compressive strength at the age of 56 days. The results showed a decrease in compressive strength of up to 20% and 24% for fly ash and limestone filler, respectively, compared to the control mix. 

Data on the residual properties of SCC containing metakaolin are scarce in the available literature. Anand et al. [22] tested SCC with the addition of silica fume, fly ash and metakaolin heated to 900 °C and cooled with air or water. They found that mixes with metakaolin exhibited the densest structure and, consequently, the highest strength reduction of up to 73% when cooled in air. In contrast, Abdelmelek et al. [23] studied the effect of high temperature up to 800 °C on high strength concrete (HSC) in which cement was replaced with 6 and 9% metakaolin. Among the tested concretes, the concrete mix with higher metakaolin content showed the best behavior in terms of residual compressive strength.

Although SCC has been used in practice for more than 30 years [24], a literature review has shown that only limited and conflicting data are available on the effect of high temperatures on high strength SCC concretes with different mineral additives. Therefore, the main objective of this work is to contribute to a better understanding on the effect of different mineral additives on the compressive behaviour of SCC after high temperature exposure. For this purpose, 10 mixes, i.e., one reference and nine mixes divided into three groups, depending on mineral additive used, were prepared and tested: metakaolin (5–15% by cement weight), fly ash (10–30% by cement weight) and limestone (5–15% by cement weight). Microstructural analysis, stress–strain relationships, compressive strength, modulus of elasticity and strain at peak stress were tested at room temperature and after high temperature exposure. To exclude the influence of aggregate decomposition on the residual properties, a Thermogravimetry/Differential Thermal Analysis (TG/DTA) of the dolomite aggregate was performed to determine the decomposition temperature. Based on the results of TG/DTA, a temperature range up to 600 °C was chosen. This paper summarizes the findings of the tests performed.

## 2. Materials and Methods

### 2.1. Constituent Materials

**Figure 1:**
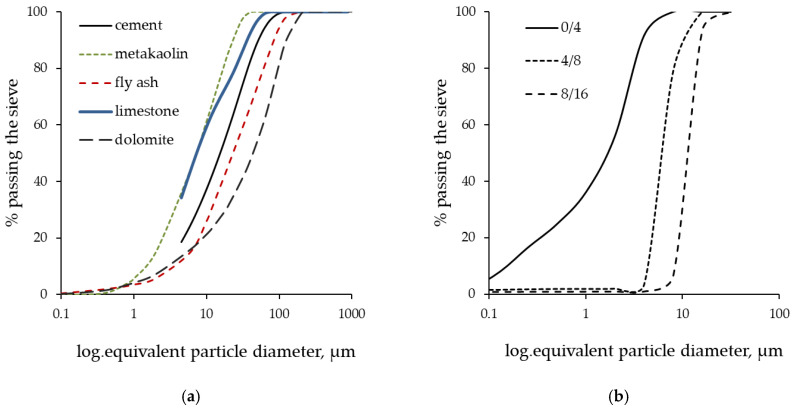
Granulometry of the (**a**) binder materials and dolomite filer, (**b**) aggregate.

The constituent materials for concrete production included the following:

1. Binder materials:
  – Portland cement CEM I 42.5R according to EN 197-1 [25] and dolomite powder were used in all mixes. The compressive strength of cement, tested according to the standard EN 196-1 [26], was 61 MPa after 28 days.
  – Mineral additives used (metakaolin, fly ash and limestone) are available on the Croatian market. Table 1 summarizes the properties of the Portland cement and mineral additives used in the studied mixes, while Figure 1a shows the particle size distribution obtained by the laser diffraction method.
2. Natural, crushed dolomite aggregate (0/4, 4/8 and 8/16 mm) with a particle size distribution is shown in Figure 1b.
3. A superplasticizer based on modified polycarboxylic ether polymers, with a relative density of 1100 kg/m^3^, and a viscosity modifying agent (VMA) based on high-molecular weight synthetic copolymer with a relative density of 1009 kg/m^3^, were added during concrete production to set the workability of the tested mixes.
4. Tap water was obtained from the general municipal drinking-water supply, which contained a negligible amount of chloride substances.

**Figure 2:**
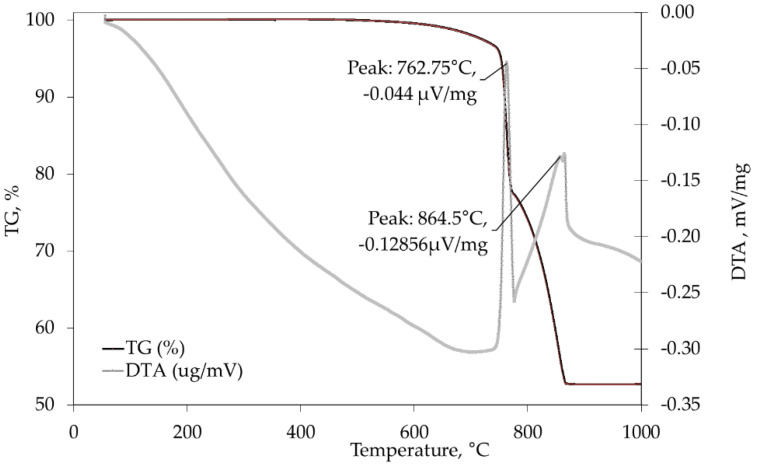
TG/DTA graph of dolomite aggregates.

The dolomite aggregates and the dolomite powder used in the concrete mixes came from the same quarry. TG/DTA analyses were performed on the dolomite aggregate to determine the temperature at which the decomposition of the dolomite occurs. The results of the analysis, presented in Figure 2, showed that their decomposition started at a temperature of 762.8 °C. After 864.5 °C, the dolomite’s mass decreased by more than 47%. 

### 2.2. Concrete Mix Design and Specimen Dimensions

The details of the studied concrete mixes are provided in Table 2. CEM I 42.5 R was used as the binder in the reference mix, M2. In the other nine mixes, M3–M11, the mineral additives, metakaolin (MK), fly ash (FA) and limestone filler (LF) were used as partial replacement for cement by weight. All concrete mixes were designed to achieve a slump flow of 700 ± 50 mm. Other fresh concrete properties required for SCC mixes were also tested to ensure good flowability, workability and segregation resistance of the tested mixes and are also listed in Table 2. All mixes met the requirements for SCC according to EFNARC [27] and EN 206-9 [28]. 

An analysis of the fresh properties of the tested mixes can be found in Jelcic Rukavina et al. [29]. All concrete specimens intended for testing were cast without compaction and vibration. After casting, the specimens were kept covered in the laboratory for 24 h until demolding to prevent the evaporation of water. Then, the specimens were placed in a curing chamber at 20 ± 2 °C and RH ≥ 95% for the next 27 days. After 28 days of curing, the specimens were kept in the laboratory (T = 15–25 °C; RH = 50–70%) until the age of one year, when heating and subsequent testing took place. In order to study the effects of high temperatures on the change in mechanical properties of SCC, the cylinders with dimensions Ø/L = 75/225 mm (i.e., with the required slenderness of 3) were prepared according to the recommendations of RILEM TC 200 HTC [30,31].

### 2.3. Heat Treatment

Heat treatment was also performed according to the recommendations of RILEM TC 200 HTC [30,31]. The specimens of all mixes were exposed to three different temperature cycles with target temperatures of 200 °C, 400° C and 600 °C in an electric furnace (capable of heating up to 1350 °C), while the specimens of reference mix, M2, were exposed to an additional cycle with a target temperature of 800 °C to investigate the effect of dolomite (aggregate and filler) decomposition on the mechanical properties of the tested mix.

**Figure 3:**
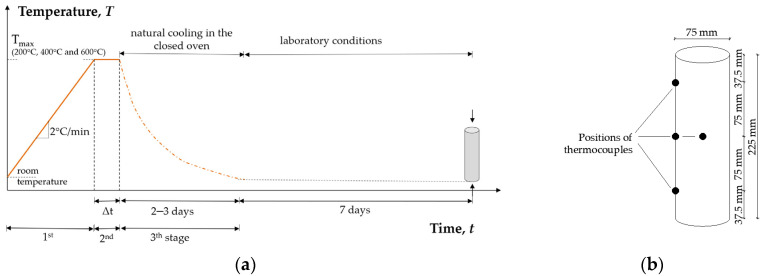
(**a**) Schematic representation of temperature cycle; (**b**) position of NiCr thermocouples in the specimens.

Each temperature cycle consisted of 3 stages (Figure 3a):

5. First stage: heating at a rate ΔT/Δt of 2 °C/min (in the furnace) up to the target temperature;
6. Second stage: keeping the target temperature constant until steady-state thermal conditions throughout the specimens were ensured, Δt;
7. Third stage: slow natural cooling to ambient temperature in a closed furnace to avoid thermal shock.

**Figure A1:**
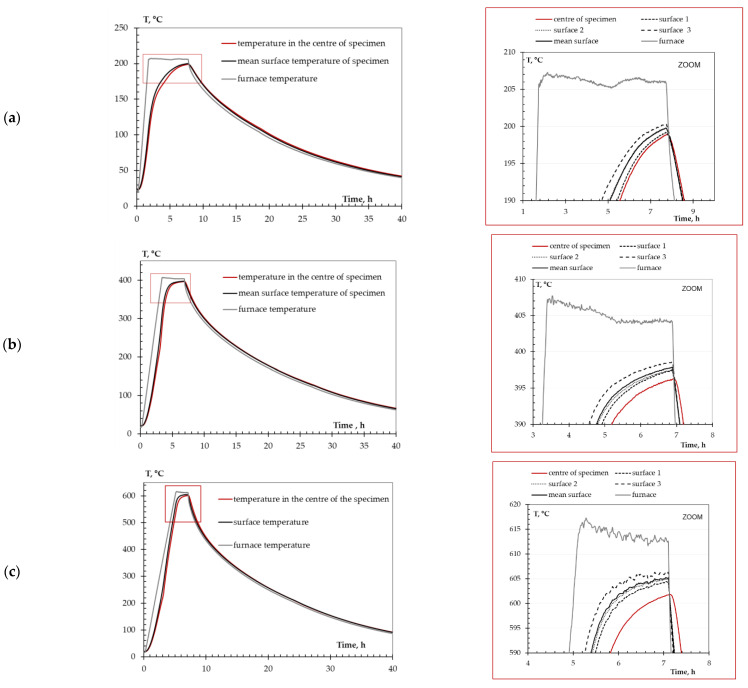
Temperature development for mix M11-LF15 during heating to target temperature of (**a**) 200 °C, (**b**) 400 °C and (**c**) 600 °C.

Temperature distribution in the specimens was monitored with 4 Ni-Cr thermocouples embedded in one specimen during each temperature cycle: three on the specimen surface at mid-height with equal spacing and one in the centre, all embedded during casting, Figure 3b, as proposed by recommendations of RILEM TC 200 HTC [31]. To achieve steady-state thermal conditions through the specimens, a preliminary analysis of the heating regimes was performed, and the time of the second stage (Δt) was determined for each target temperature. Figure A1 in Appendix A shows the monitored temperatures achieved through specimens for mix M11. A very similar temperature development was observed in the specimens from other mixes.

After cooling, the specimens were stored under standard laboratory conditions until testing, which occurred 7 days after cooling to ambient temperature, when the lowest values of residual mechanical properties were expected [32]. 

**Figure A2:**
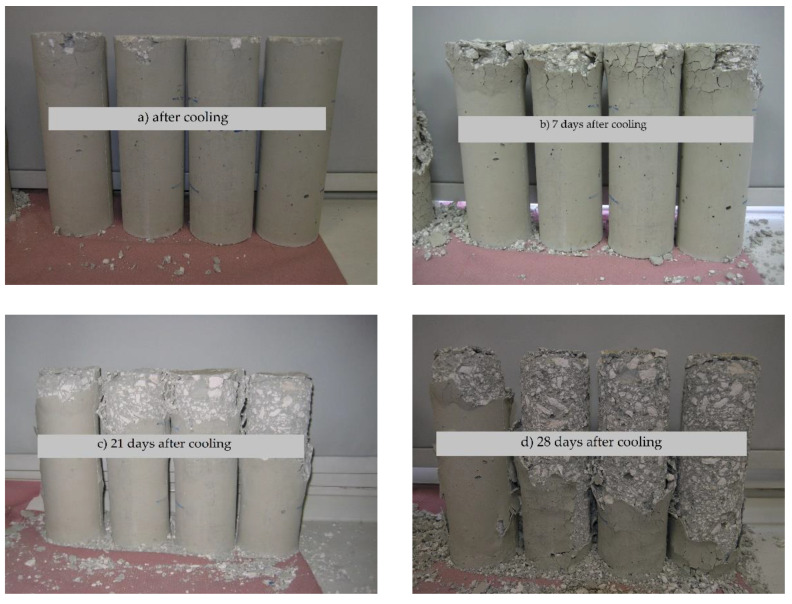
The post-cooling appearance of reference concrete specimens heated to 800 °C (**a**) after cooling, (**b**) 7 days after cooling, (**c**) 21 days after cooling, (**d**) 28 days after cooling.

The reference mix was heated to 800 °C to evaluate the effect of dolomite decomposition on post-cooling behaviour. In accordance with the TG/TDA analysis shown in Figure 2, the decomposition of the dolomite occurred at 763 °C. After cooling to room temperature, the specimens, although exhibiting a high degree of cracking, remained “in one piece” (see Figure A2 in Appendix B). However, the extent of damage that developed within the next 7 days prevented further testing. According to Liu et al. [33], this type of degradation can be regarded as post-cooling spalling after exposure to very high temperatures. Upon heating, lime, (CaO), is formed from the dehydration of portlandite, (Ca(OH)_2_), from cement matrix at temperatures between 400 and 600 °C or, in this case, from the thermal decomposition of the dolomite aggregate and dolomite filler at a temperature of 763 °C. After the specimens cooled, moisture from the air was absorbed by the heated concrete and gradually penetrated deep into the exposed specimens. The absorbed moisture caused the rehydration of the lime, which was accompanied by a volume increase of 44% [34], resulting in severe cracking and falling of parts of the concrete specimens. Therefore, a maximum temperature of 600 °C was chosen to study the influence of binders on the properties after cooling.

### 2.4. Microstructural Analysis

The morphology and microstructure before and after heat treatments of the specimens were analyzed with an electron scanning microscope (SEM, JEOL Ltd., Tokyo, Japan), type JEOL 5500 LV, connected to a spectrometer-Oxford energy dispersion spectrometer (EDS, Oxford Instruments NanoAnalysis & Asylum Research, High Wycombe, UK), using backscattered electrons and low vacuum. The parameters for the microscopic analyses were as follows: acceleration of 20 kV, working distance of 18–22 mm and working pressure of 17–28 Pa. The specimens were cut out from the central part of the specimen at a height of 5 cm. The specimens were further sawed to 1 cm and polished in the transverse direction. After sawing, the specimens were stored in isopropanol until analysis. The analysis was conducted on specimens taken from the reference mix and on one mix per group of mineral additives.

### 2.5. Compressive Stress–Strain Behaviour

**Figure 4:**
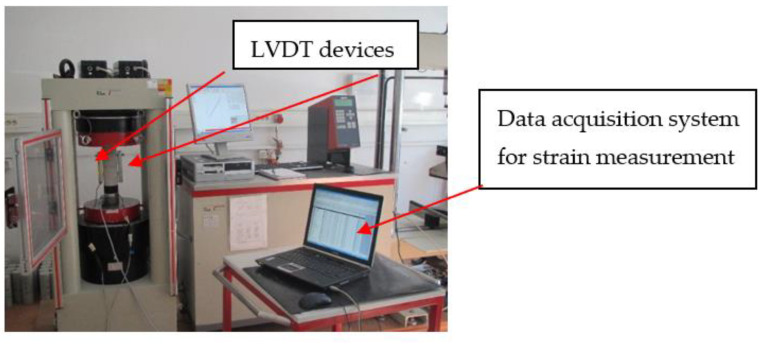
Experimental setup—test machine and electronic equipment for compressive stress–strain monitoring.

**Figure 5:**
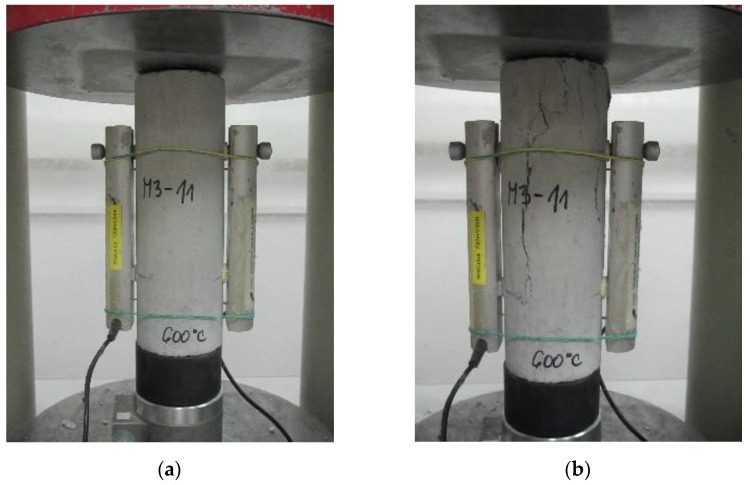
Appearance of the tested specimens (**a**) prior and (**b**) after mechanical loading.

The uniaxial compression tests were performed using a universal testing machine with a capacity of 3000 kN (Figure 4). The tests were performed at a constant displacement rate of 0.02 ‰/s. To monitor deformations during the test, LVDT gauges (Spring d.o.o., Zagreb, Croatia) with a measuring length of 10 cm were attached to the central part of the specimens. Data acquisition was performed using HBM’s MGCplus data acquisition system (MGCplus, Hottinger Brüel & Kjaer GmbH, Darmstadt, Germany). The Catman computer programme from the same manufacturer was used to process and graphically display the data. Prior to testing, additional rubber bands were attached to each specimen to hold the displacement gauges in place after the cracks appeared, since the untreated specimens and those treated at 200 °C sometimes burst explosively when the peak stress was reached (Figure 5b).

Three specimens of each concrete mix were tested per target temperature. Unheated specimens were also tested to determine the effect of heating temperature on the studied properties. The compressive strength, *f*_c_, corresponded to the peak stress (*σ*_max_) reached in the material during the test. The modulus of elasticity was calculated from the obtained stress–strain curves in the range from 10% to 1/3 of the peak stress.

## 3. Results 

### 3.1. Spalling Occurrence during Heating

**Figure 6:**
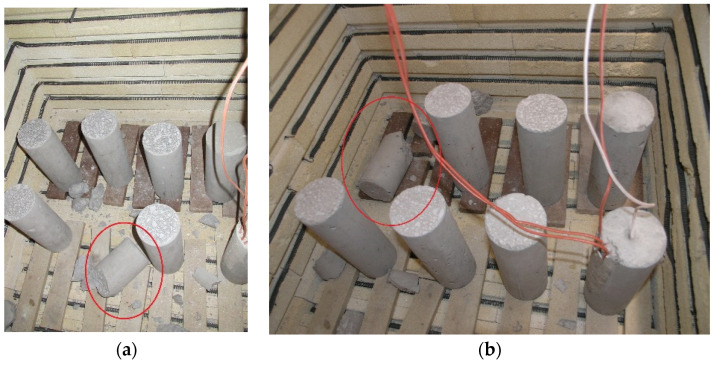
Specimens in the furnace that explosively spalled during heating to (**a**) 400 °C; (**b**) 600 °C.

Although the present study referred to concrete with high strength and dense microstructure, of all the specimens that were subjected to thermal treatment, explosive spalling occurred only in two specimens during heating to 400 °C (Figure 6a) and 600 °C, (Figure 6b), respectively. The fact that explosive spalling did not occur in the other specimens can be explained by the low moisture content, since the test was performed when the specimens were already more than a year old. In addition, residual moisture in the specimens had the possibility to evaporate not only due to the relatively small specimen dimensions but also due to the slow increase in furnace temperature of 2 °C/min. Since explosive spalling is not the subject of the present work, no further analyses of the spalled specimens were performed.

### 3.2. Microstructural Analysis

**Figure 7:**
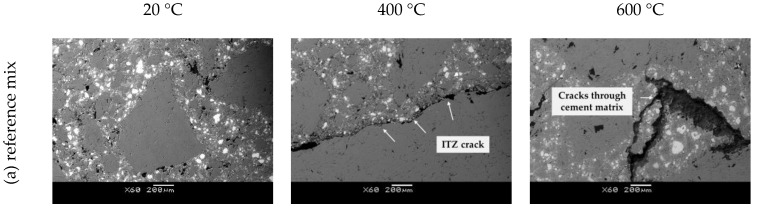

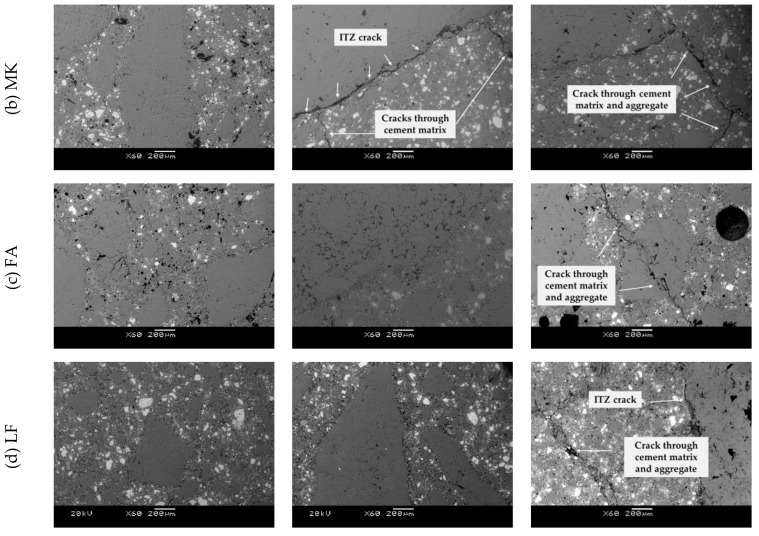
SEM images of tested mix at room temperature and after fire exposure to 400 and 600 °C for (**a**) reference mix, (**b**) mix with MK, (**c**) mix with FA and (**d**) mix with LF.

In heated concrete, the cement matrix expands slightly at temperatures between 80 °C and 100 °C, followed by significant shrinkage at higher temperatures. In contrast, the aggregate expands and forms large differential thermal stresses that can result in severe microcracking in the interfacial transition zone (ITZ) [32,34]. Since ITZ is considered as the weakest part of concrete and each mineral additive used has a different effect on its density, Figure 7 shows SEM images of the ITZ from one specimen of each group analyzed for temperatures of 20 °C, 400 °C and 600 °C. 

The analysis of the microstructure of the unheated specimens (20 °C) showed that the ITZ of each specimen can be related to the particle size of a particular mineral additive in the mix, given as Blaine fineness in Table 1. The largest Blaine fineness of the particles of MK (10,260 cm^2^/g) compared to other the binders used (8948 and 3290 cm^2^/g for LF and FA, respectively) resulted in the densest ITZ. This dense interface in the MK specimen resulted in more severe damage compared to specimens with FA and LF after temperature treatment at 400 °C. In addition to a wide crack in the ITZ, two cracks in the cement matrix can also be observed in the MK specimen. The specimen with FA, on the other hand, showed no visible cracks after treatment at 400 °C. After 600 °C, all specimens showed a similar appearance, with cracks appearing in both the cement matrix and the aggregate.

### 3.3. Compressive Stress–Strain Relationship

**Figure 8:**
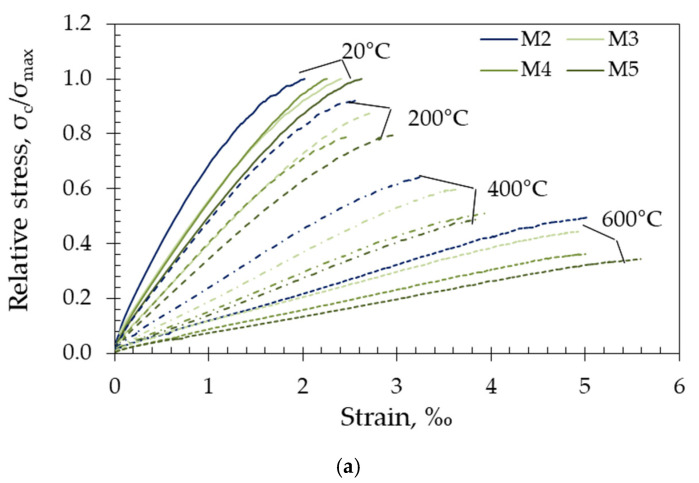

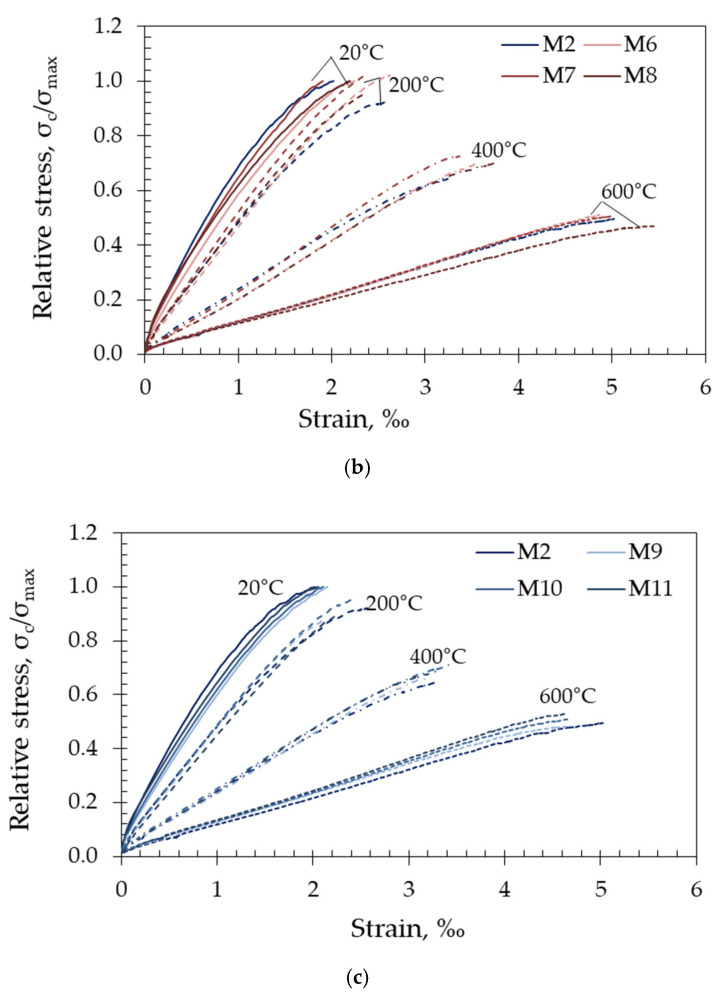
Normalised stress–strain curves for (**a**) metakaolin based (M3–M5), (**b**) fly-ash based (M6–M8) and (**c**) limestone based (M9–M11) in relation to the reference mixture (M2) as a function of temperature.

**Figure A3:**
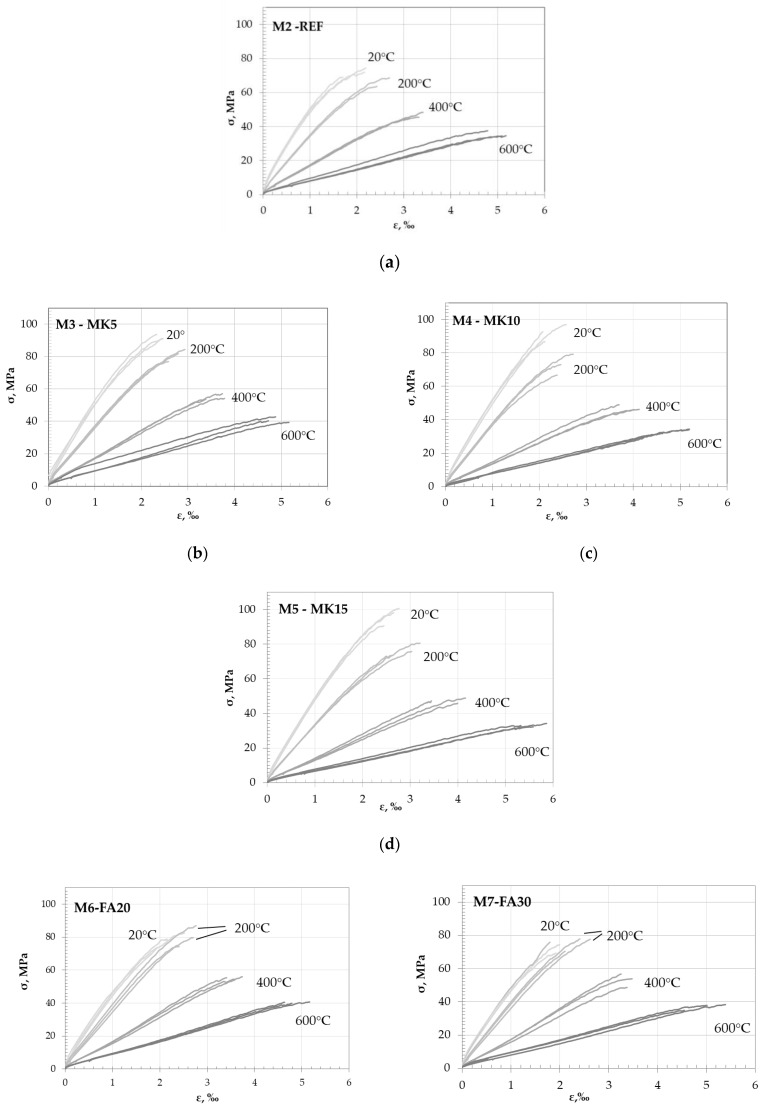

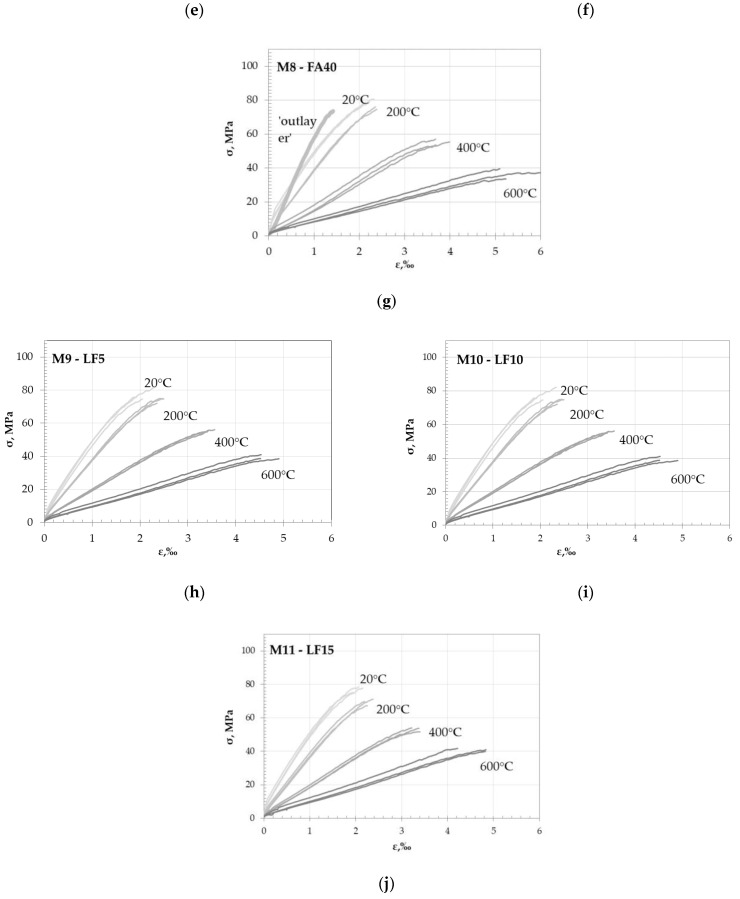
Stress–strain curves of the tested mixes: (**a**) reference mix; (**b**–**d**) mixes with metakaolin; (**e**–**g**) mixes with fly ash; (**h**–**j**) mixes with limestone.

Figure 8 shows normalized stress–strain curves as a function of temperature for each target temperature. Separate stress–strain curves for each tested specimen are provided in Figure A3 in Appendix C. 

The curves shown include an ascending branch up to the peak stress. The descending portion of the σ-ε curve was not recorded during the test because the rate of displacement was controlled by the piston movement of the test machine.

As observed from the curves given in Figure A3 in Appendix C, good reproducibility of the results was achieved after they were exposed to the respective target temperature. Due to the more homogeneous microstructure compared to conventional concrete, the stress–strain curves of the tested concrete are rather linear compared to conventional vibrated concrete. In addition, the shape of the curves was strongly influenced by temperature. As temperature increased, the curve became smoother, with lower maximum stress and higher deformation at peak stress. This can be applied for all mixes except those with fly ash after exposure to 200 °C, where the curves of the heated specimens were very close to those of the unheated ones (Figure 8b).

Comparing the stress–strain curves shown in Figure 8 within each tested group, it can be observed that the different amounts of FA (mixes M6–M8) and limestone (M9–M11) in the concrete mixes resulted in similar behaviours, while the mixes with different MK content (M3–M5) showed larger variations after all target temperatures.

As mentioned above, the compressive strength (peak stress), static modulus of elasticity and strain at peak stress were determined from the stress–strain curves and analyzed in the next sections.

### 3.4. Peak Stress—Compressive Strength

**Figure 9:**
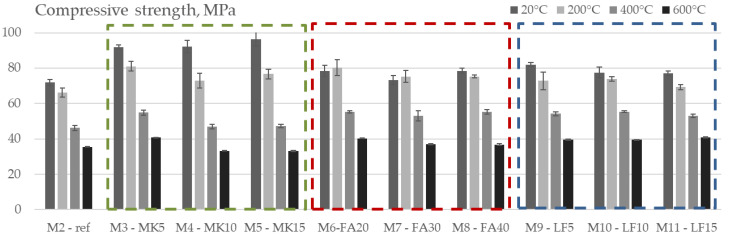
Compressive strength vs. temperature of tested mixes.

Figure 9 shows the peak stress or compressive strength, of unheated SCC specimens (20 °C) and specimens heated to all target temperatures (200 °C, 400°C and 600 °C). The initial compressive strength ranged from 71.8 MPa for M2 to 96.5 MPa for M5, i.e., the difference is up to 34%.

It can be observed from the figure that all the mineral additives used (both pozzolanic and non-pozzolanic) had a positive effect on the compressive strength of the unheated specimens. The highest values after one year of curing were obtained for SCC concrete mixes in which cement was replaced by metakaolin (mixes M3–M5). Compared to the reference mix, the compressive strength of the mixes with MK increased with an increase in the content of MK up to 34%. The FA mixes increased the compressive strength of the reference mix by up to 9.3%, and the addition of limestone filler increased it up to 14%. The positive effect of MK and FA on the development of strength, especially at later ages, can be attributed to the effect of the filler in combination with the acceleration of cement hydration and the pozzolanic reaction with calcium hydroxide [35]. 

**Figure 10:**
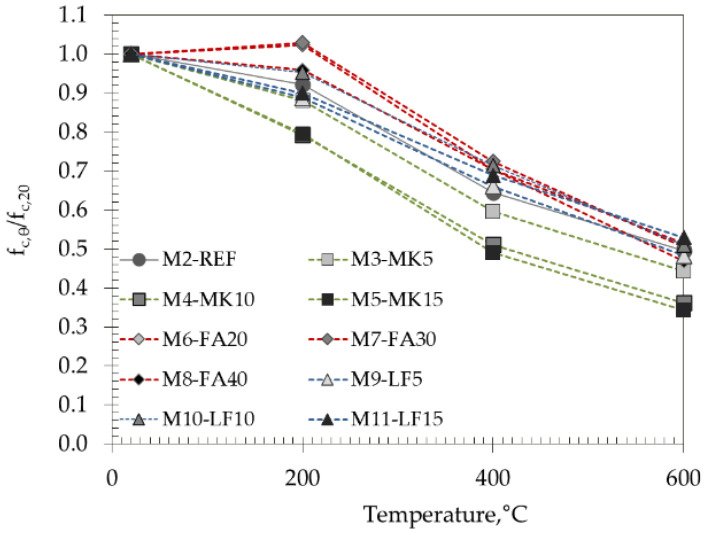
Normalized values of compressive strength vs. temperature.

To illustrate the effect of high temperatures on the change in compressive strength, Figure 10 shows the relative values that clearly indicate the influence of the specific mineral additive on the residual compressive strength of the tested mixes. The deviations between all the mixes at the defined target temperatures were up to 24% depending on the additive used. 

The negative effect of the addition of MK is evident at all treatment temperatures. The higher the percentage of MK in the concrete mix, the lower the relative residual compressive strength. For the M5 mix, the strength loss after a temperature of 200 °C, 400 °C and 600 °C was about 21%, 51% and 66%, respectively. Compared to the reference mix, the decrease in compressive strength was higher by 13–15%. As mentioned above, the negative effect of MK addition on the residual strength is a result of the denser microstructure shown in Figure 7, especially in ITZ, where pronounced cracks appeared at temperatures after heating to 400 °C. When HSC was tested with 5%, 10% and 20% of MK, Poon at al. [34] obtained a different pattern, i.e., an increase in concrete strength of 2–8% was observed after heating to 200 °C, while a slight decrease of 5–8% occurred after 400 °C, which is similar to results obtained by Abdelmelek et al. [23] who tested SCC with MK. 

The replacement of cement with FA in an amount of 20–40% had a positive effect on the residual compressive strength. After exposure to 200 °C, a slight increase (up to 4%) in compressive strength was observed in mixes with FA compared to their strength at 20 °C. In addition, the relative compressive strength of the mixes with FA was 13% higher compared to the reference mix. This can be explained by the subsequent hydration of the unhydrated cement particles due to internal steam curing due to the hot steam from the internal autoclave condition or the subsequent pozzolanic reaction of fly ash and calcium hydroxide due to the free movement of moisture in the material due to the high temperature [36]. In addition, microstructural analysis showed that the ITZ of FA mixes was not as dense as that of MK (Figure 7b). 

Mixes with limestone (as a non-pozzolanic material) showed comparable behaviour to the reference mix over the entire temperature range, with variations within 5%.

In accordance with Abed et al. [17], the residual compressive strength with increasing temperature can be divided into five main stages: (1) room temperature at 50 °C, characterized by a moderate decrease in compressive strength due to the loss of free water; (2) 150–300 °C, characterized by a constant or slightly increased compressive strength as a result of C-S-H rehydration; (3) 300–400 °C, characterized by an approximately constant or slightly decreased compressive strength due to the breakup of the chemically bound water; (4) 400–800 °C, characterized by a drastic decrease in compressive strength; (5) >800 °C, characterized by the complete loss of concrete strength. Although different temperature increments were chosen in this study, the obtained results showed that major strength loss occurred earlier up to a temperature of 400 °C.

**Figure 11:**
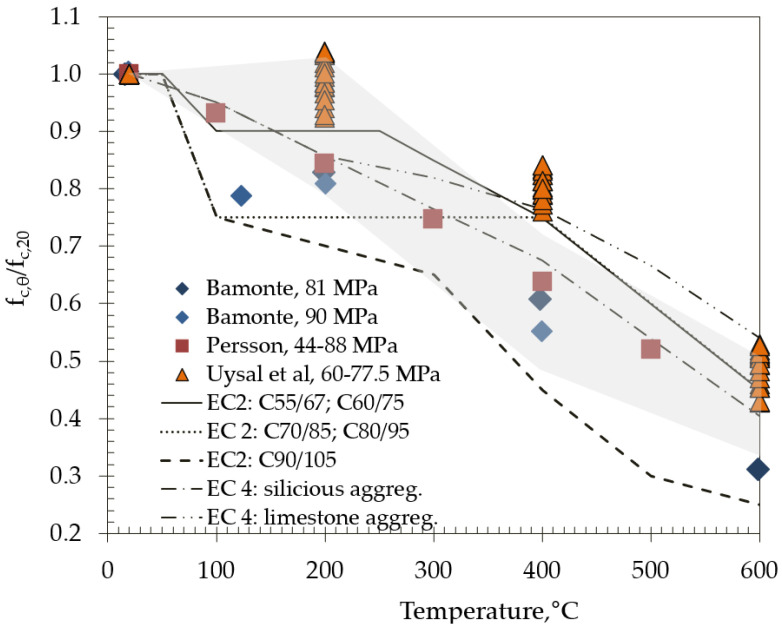
Normalized values of compressive strength compared to results of other studies and EC2 and EC 4.

The recommended values provided in Eurocodes EC2 [5] and EC4 [6] for the reduction in concrete compressive strength due to exposure to high temperatures for both normal strength and high strength concretes are shown in Figure 11 and compared with the results obtained in this work (gray shadow area in Figure 11). 

EC2 [5] recognizes that a greater reduction in strength is required for high strength concrete. Mixes M3–M11 could be classified in strength class C70/85 or higher, according to their 1-year strength; thus, it can be observed from Figure 11 that EC2 would overestimate the strength reduction for these mixes at a temperature of 200 °C. Mix M5 with 15% of metakaolin meets the requirements of strength class C90/105, and it can be seen from Figure 11 that EC2 also overestimates strength reduction due to the high temperature.

Figure 11 also makes comparisons with studies on high strength SCC concrete by Persson [37] and Bamonte et al. [38]. These studies were chosen since the initial strength of tested concrete mixes are similar, although different aggregate, natural, round river gravel and crushed gneiss and gravel, respectively, were used, which may affect the results. As observed from the figure, their results agree well with the compressive strengths obtained with the mixes with MK. 

With the exception of MK, the obtained results after 200 and 600 °C agree well with the results of Uysal et al. [39] but differ after treatment at 400 °C. Any discrepancies in the results obtained may be due to different curing and heating durations.

### 3.5. Modulus of Elasticity

**Figure 12:**
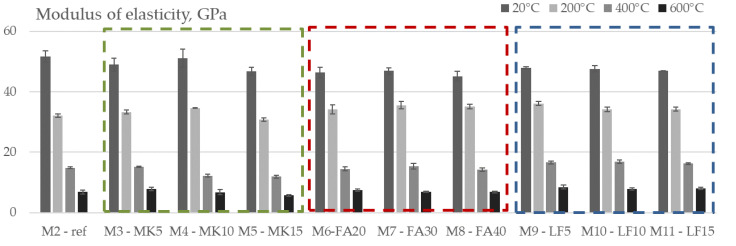
Modulus of elasticity vs. temperature of tested mixes.

**Figure 13:**
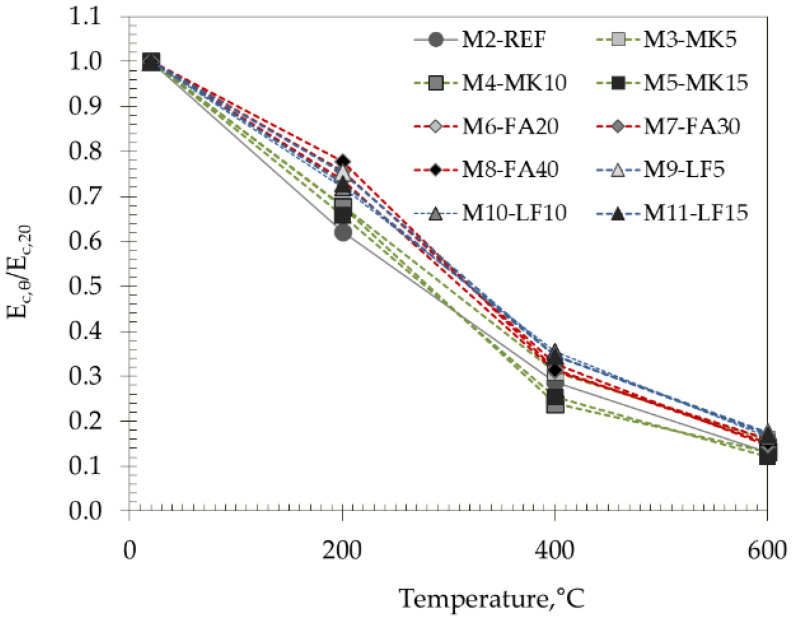
Relative values of modulus of elasticity with increasing temperature.

High temperatures have a negative effect on the elastic properties of concrete, mainly due to the decomposition of hydrated cement products and the breaking of bonds in the microstructure [40]. Figure 12 and Figure 13 show the effects of high temperatures on the residual modulus of elasticity of the SCC concretes tested, both in absolute and relative values.

The initial values of modulus of elasticity ranged from 45.1 GPa for M8 to 51.7 GPa for the M2 mix (i.e., the difference is around 14%) and decreased almost linearly with temperature.

As expected, a greater effect of elevated temperatures was observed on the modulus of elasticity compared to the compressive strength, but in this case, the differences between the studied mixes were smaller. The highest difference in relative modulus of elasticity between the studied mixes is 14% after 200 °C and continues to decrease with increasing temperature. After 600 °C, the difference between mixes was 6%.

Considering the different groups of studied mixes, a very similar pattern as for compressive strength was obtained—the lowest relative values after 400 °C and 600 °C were obtained within MK-based mixes, i.e., the initial modulus of elasticity of M5 mix decreased for 75% and 88%, respectively. After 200 °C, the reference mix had negligibly lower values compared to MK mixes—decrease was around 38%. FA and LF had a beneficial effect on the residual modulus of elasticity compared to reference mix. As with the compressive strength, there was a strong loss of the modulus of elasticity up to 400 °C in all tested mixes. 

### 3.6. Peak Strain

**Figure 14:**
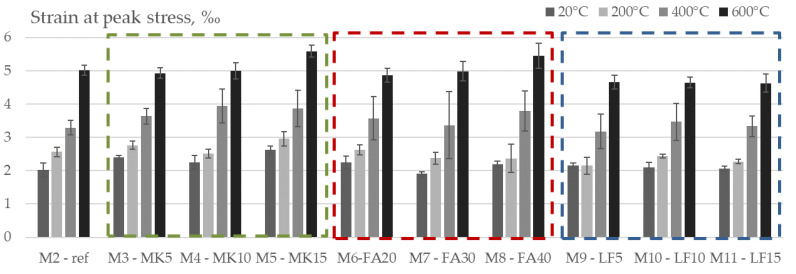
Strain at peak stress vs. temperature of tested mixes.

**Figure 15:**
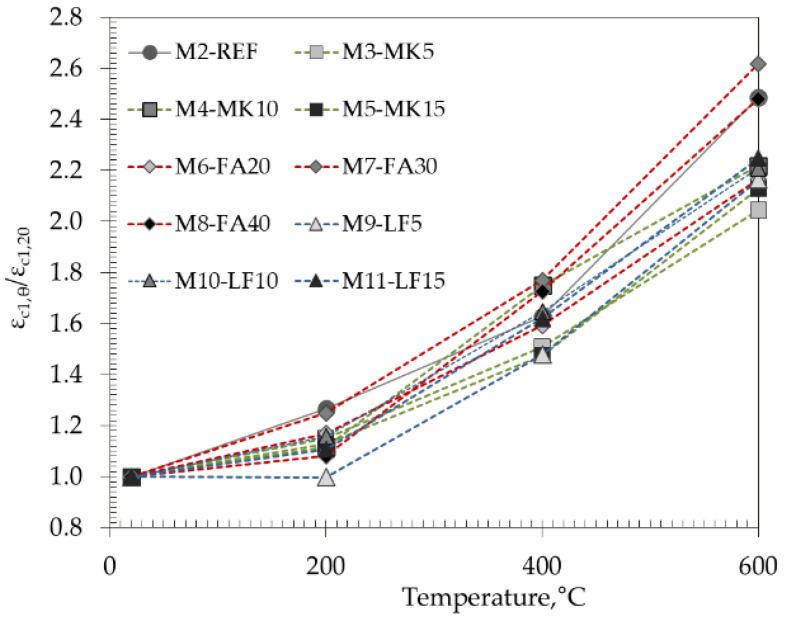
Relative values of strain at peak stress with increasing temperature.

Figure 14 and Figure 15 show the peak strain, which is the strain corresponding to the ultimate stress applied for the studied concrete mixes with increasing temperature (200 °C, 400 °C and 600 °C) in both absolute and relative values. The initial peak strain of the tested mixes varied from 1.90‰ for M7 up to 2.62‰ for M5. 

In accordance with the observations of Bamonte et al. [38] and Chang et al. [40], the peak strain increases with temperature, indicating a higher ductility of concrete at elevated temperatures. The highest increase after heating to 600 °C was obtained in FA mixes with 30 and 40% cement replacement, which was up to 2.6 times. Opposed to compressive strength and modulus of elasticity, peak strain seems to exhibit a higher degree of variability.

## 4. Modelling of Stress–Strain Behaviour

The prediction of the stress–strain relationship of concrete subjected to uniaxial compression at room temperature has been a topic of research papers for many decades [41,42,43]. Recently, several researchers performed experimental investigations of stress–strain relationships of concrete during or after exposure to fire [40,44,45]. Different equations have been proposed to describe the stress–strain diagram of concrete. One of the simplest models was proposed by Popovic (Equation (1)):

(1) $$\frac{\sigma }{\sigma_{\max}}=\frac{\varepsilon }{\varepsilon_{\max}}\frac{\beta }{\beta -1+{\left(\frac{\varepsilon }{\varepsilon_{\max}}\right)}^{\beta }}$$

where *σ* is the compressive stress, *σ*_max_ is the peak stress (equivalent to *f*_c_), *ε* is strain, *ε*_max_ is the strain at peak stress and *β* is parameter describing the curvature of the stress–strain relationship [41,46]. It was found that the Popovics model can accurately describe the stress–strain relation for concrete after being exposed to temperatures up to 500 °C while at temperatures above 500 °C, the concave-up part of the stress–strain curve can form at low stresses [40,45]. To provide a better fit to the experimental data, especially to the descending branch of the stress–strain relationship, several modifications of Equation (1) have been proposed by introducing additional correction coefficients into the equation [40,45,47].

**Figure 16:**
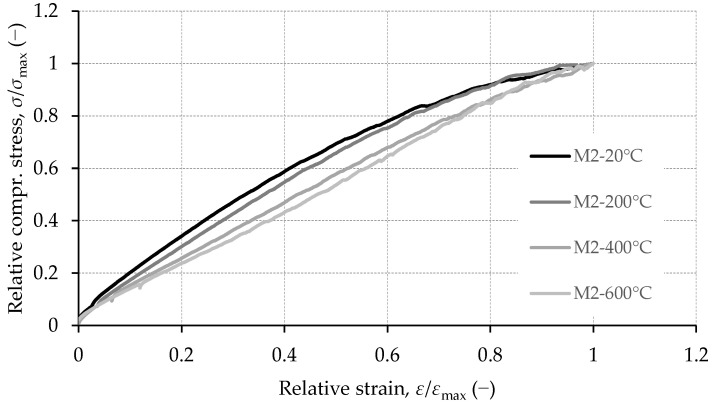
Relative compressive stress vs. relative strain for mixture M2.

As said previously, increasing temperature changes curvature of the stress–strain relationship. An example of the influence of high temperature treatment on the shape of stress–strain relation in concrete is shown in Figure 16. As temperature increases stress–strain relationship shifts from curved towards linear. This was confirmed on all mixes analyzed within this research and is consistent with the data presented in the literature [40,44,45]. The shape of the stress–strain curves of concrete under uniaxial compression is explained by progressive microcracking [46]. Up to about 50% of compressive strength microcracking is mainly concentrated at the ITZ between aggregate and cement matrix. A further increase in stresses causes cracks to form in the matrix, which produces more pronounced deviations of the stress–strain curve from linearity. In concrete exposed to elevated temperature, cracks were already formed in the matrix and part of the aggregated before any load was applied. SEM imaging shows that the amount of matrix and aggregate cracking increases with temperature. These additional in-built cracks change the interaction between failure mechanisms under compressive loads, which is reflected on the shape of the stress–strain curve.

**Figure 17:**
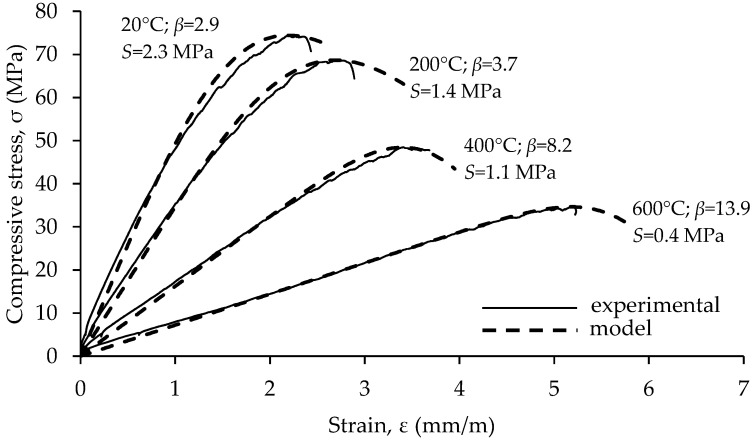
Comparison between model and experimental values.

Nonlinear regression analysis using least squares method was applied to evaluate how well the model from Equation (1) represents experimentally determined stress–strain curves. Multiplying Equation (1) with peak stress (*σ*_max_) yields the stress–strain relationship in absolute units. The differences between the values predicted by the model and the observed values were evaluated by the root mean square error (*S*). The range of *S* for samples from all mixes kept at 20 °C was 1.8–3.9 MPa. For specimens exposed 200 °C, 400 °C and 600 °C, the range of *S* was 0.9–1.9 MPa, 0.2–1.3 MPa and 0.2–1.2 MPa, respectively. As an example, a comparison between model and experimental values for specimens from mix M2 is presented in Figure 17.

**Figure 18:**
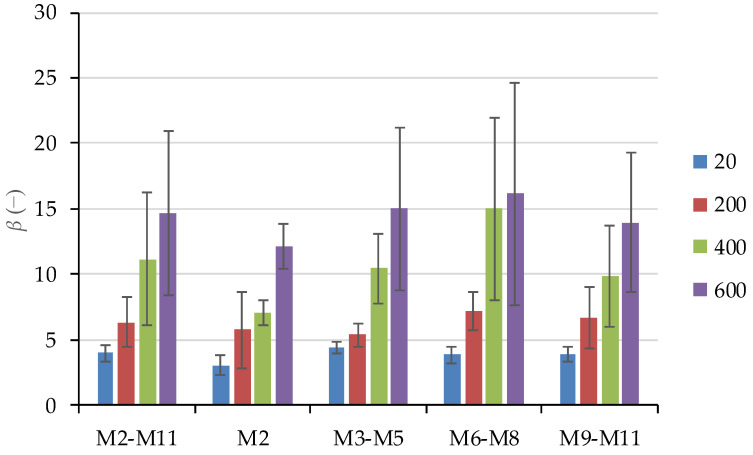
Parameter *β* determined by regression analysis to experimentally determine stress–strain relationships.

Equation (1) contains only one unknown parameter (*β*), which is determined by regression analysis. *Β* represents the ratio *E*_0_/(*E*_0_ − *E*_p_), where *E*_0_ is the initial tangent Youngs modulus and *E*_p_ is secant Youngs modulus at peak stress [41]. Since an increase in temperature shifts the stress–strain curve toward a linear shape, the difference *E*_0_ − *E*_p_ should decrease and consequently *β* increase. This was confirmed by analyzing *β* for specimens from mixes M2–M11. As temperature increases, parameter *β* increases, indicating that the stress–strain curve approaches linear dependence. Figure 18 compares the average values of *β*, and its standard deviations are compared between concrete mixes with different mineral additives. Increasing temperature also increased the dispersion of *β*.

It is important to emphasize that the variation of *β* has a significantly different effect on the shape of the stress–strain curve for low and high values of *β*. One method of quantifying the effect of *β* on the shape of a stress–strain curve is the use of the area under the stress–strain curve, which represents the mechanical energy per unit volume. Equation (1) represents relative stress (*σ*/*σ*_max_) expressed as a function of relative strain (*ε*/*ε*_max_). The area under relative stress vs. relative strain curve is proportional to the mechanical energy consumed by deforming the material. If *β* = 1, then Equation (1) produces *σ*/*σ*_max_ = 1, which represents perfectly plastic material; on the other hand, if *β* proceeds to infinity, Equation (1) becomes *σ*/*σ*_max_ = *ε*/*ε*_max_ and represents linear elastic material. Integrating Equation (1) in the range of relative strain between 0 and 1 provides an area of 1 for *β* = 1 and 0.5 for *β* → ∞. The values of *β* for concrete start at 1.3 or 1.4 [40,44]. By analyzing the dependence of the area (*A*) under the relative stress–strain curve, the analysis shows that the area decreases with increasing *β* approximately according to *A* = 0.5 (*β^−k^* + 1), where *k* ≈ 1.095. Using this equation for *A*, it can be shown that a change in *β* within the range of 2–4 would cause approximately the same change in the area as a variation in the range of 3–17. 

**Figure 19:**
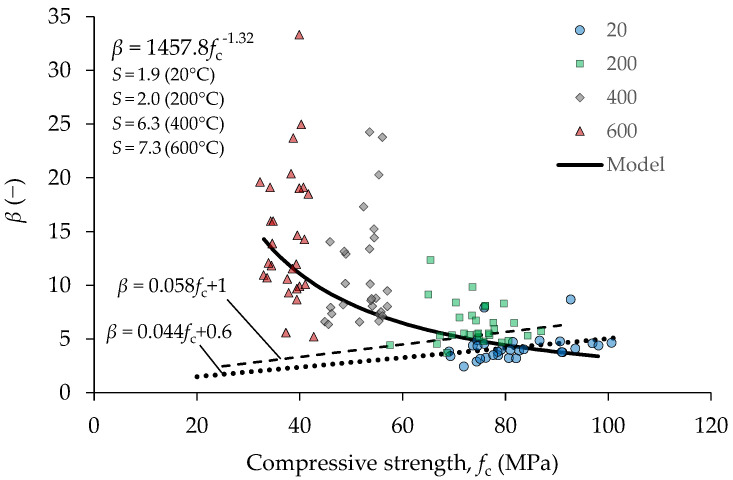
Correlation between parameter *β* and compressive strength for mixtures M2–M11.

For normal concrete cured at room temperature, Popovics proposed that *β* can be estimated from the compressive strength as 0.058*f*_c_ + 1. In this work, for specimens stored at 20 °C, the relation between *f*_c_ and *β* determined by linear regression is 0.044*f*_c_ + 0.6. Both equations have a similar slope and are compared in Figure 19. This implies that the order in which microcracking evolves during loading in the high strength concrete tested in this research is similar to that in normal strength concrete. 

The relationship between *f*_c_ and *β* for concrete exposed to elevated temperature does not follow the same trend as for concrete stored at room temperature. The correlation between *f*_c_ and *β* was analyzed for each mix separately using linear regression. It was found that power law equation provided lower errors of estimation compared to the linear model. Due to the wide dispersion of data, no significant differences between models for mixes containing different mineral admixtures were found; thus, only overall regression models based on the results of all tested samples are provided in Figure 19. The differences between values predicted by model and the observed values are evaluated by root mean square error for each temperature of exposure and are also provided in Figure 19. Although the variation of *β* increases with temperature, it can be shown that the effect of this variation on the area under the relative stress–strain curve is the greatest at low temperatures.

## 5. Conclusions

Previous studies on SCC have shown that the advantages of this type of concrete at room temperature over conventional concrete are its improved internal structure, which is achieved by more effective particle packing. Compared to normal vibrated types of concrete, a higher percentage of fines and, in most cases, mineral additives are used. In this study, new experimental results on mechanical properties were obtained by investigating the effects of elevated temperatures on the performance of high strength SCC with the common mineral additives, i.e., metakaolin, fly ash and limestone filler in different proportions. Based on the results obtained, the following conclusions can be drawn:

8. By comparing the stress–strain curves within the mixes containing the same mineral additive, it can be observed that the different amounts of FA (20–40%) and limestone (5–15%) in the studied concrete mixes lead to a similar behaviour, while the mixes with different MK content (5–15%) show larger variations after all target temperatures.
9. The different mineral additives used in this study (MK, FA and LF) affected the development of different microstructures of the concrete, especially ITZ, which, in turn, affected the variations in the residual compressive strength by 24% and the peak strain by 38%, while the variation in the residual elastic modulus was 14%.
10. Contrary to the findings in the literature, which show that concrete retains most of its strength up to 400 °C, in this study, a significant loss of mechanical properties (compressive strength and elastic modulus) and an increase in peak strain were observed up to a temperature of 400 °C.
11. However, comparing the obtained results with the recommendations for compressive strength given in EC 2 for HSC, it can be concluded that the strength loss of EC 2 in the case of SCC with used mineral additives is too conservative in the lower temperature range (400 °C), especially for mixes containing fly ash.
12. The Popovic model for the relationship between stress and strain, developed essentially for conventional concrete, provided a good approximation relative to the experimentally determined stress–strain curves at different temperatures.

In this study, one mineral additive was added to the mix. To increase the sustainability of the concrete, the tests should be extended to binary and ternary mixes of mineral additives and their mutual interaction should be evaluated after exposure to high temperature. 

## Figures and Tables

**Table 1:** Properties of binder materials.

Property/Components	Portland Cement	Dolomite Filler	Metakaolin	Fly Ash	Limestone
Chemical analysis, %					
CaO	60.23	30.38	0.55	4.21	54.05
SiO_2_	19.81	0.00	53.53	51.87	0.66
Fe_2_O_3_	2.71	0.18	1.17	9.22	0.12
Al_2_O_3_	5.38	0.31	41.18	24.46	0.15
MgO	2.87	21.84	0.36	1.83	1.01
Na_2_O	0.8	0.05	0.08	0.23	0.08
K_2_O	0.77	0.02	0.83	1.14	0.02
SO_3_	3.07	0.05	0.08	0.56	0.09
Loss of ignition, LOI	4.47	47.58	1.36	0.54	43.87
Physical properties					
Specific gravity, g/cm^3^	3.05	2.86	2.68	2.34	2.63
Blaine fineness, cm^2^/g	3290	1630	10,260	3070	8948

**Table 2:** Mix design and properties of studied SCC mixes.

			Metakaolin			Fly Ash			Limestone		
Mix ID		M2	M3 (MK5)	M4 (MK10)	M5 (MK15)	M6 (FA20)	M7 (FA30)	M8 (FA40)	M9 (LF5)	M10 (LF10)	M11 (LF15)
Cement, kg		450	427.5	405	382.5	360	315	270	427.5	405	382.5
MK	% c.w.	-	5	10	15	-	-	-	-	-	-
	kg	-	22.5	45	67.5	-	-	-	-	-	-
FA	% c.w.	-	-	-	-	20	30	40	-	-	-
	kg	-	-	-	-	90	135	180	-	-	-
LF	% c.w.	-	-	-	-	-	-	-	5	10	15
	kg	-	-	-	-	-	-	-	22.5	45	67.5
Dolomite filer, kg		220									
Water, L		180									
v/c		0.40	0.42	0.44	0.47	0.50	0.57	0.67	0.42	0.44	0.47
Fine aggregate, kg		862	862	862	862	862	862	862	862	862	862
Coarse aggregate, kg		696	696	696	696	696	696	696	696	696	696
Superplasticizer, L		5.6	4.5	5.2	6.3	4.1	3.6	3.4	5.0	4.1	3.9
WMA, L		0.7							1.0		
Fresh concrete properties											
Density in fresh state, kg/m^3^		2499	2485	2482	2488	2462	2438	2419	2489	2486	2480
Air content, %		1.9	2.1	2.4	2.1	2.3	2.0	2.3	2.1	2.2	2.2
Slump flow		732	720	725	727	720	723	725	720	718	717
Slump flow time (t_500_), s		2.08	2.35	2.19	2.10	1.66	1.47	1.40	1.38	1.49	1.65
L-box (h2/h1), -		0.94	0.93	0.82	0.82	0.84	0.87	0.92	0.94	0.91	0.85
Segregation resistance		5	8	10	10	5	5	8	7	4	3

## Data Availability

Not applicable.

## Appendix A. Monitored Temperatures in Concrete Specimens

Figure A1 presents temperature development within concrete specimens.

## Appendix B. Dolomite Decomposition after Temperature of 800 °C

In Figure A2., the degradation of specimens is presented with elapsed time up to 28 days after cooling.

## Appendix C. Stress–Strain Curves of Tested Mixes

Figure A3 presents stress–strain curves obtained on three specimens from each mix and after each temperature cycle considered.
